# Human sperm KSper is physiologically activated by intracellular pH alkalization and CatSper-mediated Ca^2+^ signaling

**DOI:** 10.1016/j.jbc.2025.110752

**Published:** 2025-09-22

**Authors:** Hang Kang, Huafeng Wang, Jie Wu, Jiali Zhang, Xiaoning Zhang, Xuhui Zeng

**Affiliations:** 1 Institute of Reproductive Medicine, Medical School, Nantong University, Nantong, Jiangsu, China; 2 Institute of Life Science and School of Life Science, Nanchang University, Nanchang, Jiangxi, China

**Keywords:** human sperm–specific K^+^ channel, hKSper, membrane potential, pH alkalization, Ca^2+^ signaling, sperm-specific Ca^2+^ channel, CatSper, resting [Ca^2+^]_i_ level

## Abstract

The hyperpolarization of membrane potential (*V*_m_) generated by the activation of the sperm-specific K^+^ channel (KSper) is considered as an important indicator for the evaluation of sperm-fertilizing capacity. However, owing to the relatively low pH sensitivity and low Ca^2+^ affinity of human KSper (hKSper), whether the changes of intracellular pH or cytosol Ca^2+^ ([Ca^2+^]_i_) in response to physiological stimuli are sufficient to potentiate native hKSper is fairly obscure. Here, by utilizing quantitative *V*_m_ fluorometry and current-clamp recordings on human sperm, our results reliably demonstrated that physiologically relevant extracellular pH alkalization activated hKSper and evoked ∼20 mV hyperpolarization of *V*_m_. Given that sperm Ca^2+^ influx is primarily mediated by the sperm-specific Ca^2+^ channel (CatSper), fluorometric results showed that progesterone, a physiological agonist of CatSper, remarkably hyperpolarized *V*_m_ in a dose-dependent manner. This hyperpolarizing effect (∼10−15 mV estimated by population *V*_m_ measurements) was largely suppressed by pharmacological inhibition of hKSper or the loss of functional CatSper. Surprisingly, electrophysiological recordings failed to detect progesterone-elicited hyperpolarization of *V*_m_ when employing Ca^2+^ chelator-free pipette solution. However, ∼10 mV hyperpolarization could be detected when the Ca^2+^ chelator contained in the pipette. In addition, [Ca^2+^]_i_ alteration under 100 nM could potently enhance hKSper activity, suggesting that hKSper can sense resting [Ca^2+^]_i_ levels. Taken together, our results clearly illustrate the activating effect of intracellular pH and [Ca^2+^]_i_ elevation on hKSper under physiological conditions, and moreover, broaden the understanding of the Ca^2+^-involved regulatory mechanism of hKSper.

Accompanied by the entrance from the epididymis into the female reproductive tract, ejaculated sperm have to undergo various biochemical transformations to become capable of fertilization (1, 2, 3). During this process, sperm are exposed to the changeful physiological environment, which therefore induces intracellular pH (pH_i_) alkalization (4, 5), membrane potential (*V*_m_) hyperpolarization (6, 7, 8, 9, 10), and Ca^2+^ elevation (11, 12, 13). Among these cellular changes, the hyperpolarization of *V*_m_ is one of the crucial events since it is suggested to be essential for the regulation of sperm physiological functions, including capacitation, flagellar asymmetrical beat called hyperactivation, and acrosome reaction (10, 14, 15, 16). Based on the clinical examination, it was shown that human sperm collected from patients who attended *in vitro* fertilization or intracytoplasmic sperm injection treatment exhibited a depolarized *V*_m_ compared with normal sperm (17). Further reports found that the hyperpolarization extent of *V*_m_ in capacitated sperm was positively correlated with the *in vitro* fertilization success ratio (8, 9). These findings indicated that sperm hyperpolarized *V*_m_ was of significance for successful fertilization.

In mouse sperm, previous studies have shown that the regulation of *V*_m_ was primarily dominated by a sperm-specific potassium channel (mouse KSper [mKSper]) (18, 19). Electrophysiological recordings on mouse sperm revealed that intracellular alkalization was the main inducement to amplify mKSper current (18). Benefiting from the generation of knockout models, mouse slowpoke homologous 3 (mSLO3) was verified as the pore-forming subunit responsible for mKSper (19, 20). In mouse sperm lacking mSLO3, the outward K^+^ current was remarkably reduced, which, in turn, gave rise to the depolarization of *V*_m_ (19, 20). Moreover, it was found that mouse leucine-rich-repeat-containing protein 52 (mouse LRRC52) functioned as the auxiliary subunit of mSLO3 and modulated the voltage-sensitive activation of mKSper (21). Both *Slo3*^*−/−*^ and *Lrrc52*^*−/−*^ male mice exhibited severe fertility impairments, indicating the essential role of mKSper in sperm fertility (19, 20, 21).

Consistent with mouse sperm, an outwardly rectified K^+^ current could be recorded from the flagellum of human sperm (22, 23). Recently, a highly selective inhibitor of human SLO3 (hSLO3), termed VU0546110, completely blocked human KSper (hKSper), supporting that hKSper was also mediated by hSLO3 (24). Furthermore, two reports found that the genetic mutations of *SLO3* gene in human sperm resulted in the failure of the acrosome reaction and induced male infertility, which likely arose from the abnormality of *V*_m_ regulation (25, 26). Although there was no report clarifying the role of human LRRC52 (hLRRC52) on male fertility, by generating the antibody specifically targeting hLRRC52, our previous work suggested that hLRRC52 participated in regulating hKSper-controlled *V*_m_ and sperm functions (27). Collectively, these findings potently manifested the importance of hKSper activity on human sperm function.

Although the molecular components of hKSper have been basically elucidated, the regulatory mechanism of hKSper, especially under physiological conditions, remains obscure. Electrophysiological recordings have revealed a less prominent pH sensitivity of hKSper compared with its murine counterpart, mKSper (22, 28). Extracellular pH (pH_e_) elevation that occurs during the whole fertilization process may serve as a potential physiological trigger for hKSper activation (5, 18, 29). Intriguingly, hKSper also evolved a distinct Ca^2+^-dependent characteristic (22, 23). Artificial intracellular Ca^2+^ ([Ca^2+^]_i_) elevation to 50 μM prominently activated hKSper (23). Given that sperm-specific Ca^2+^ channel (CatSper) is regarded as the primary Ca^2+^ channel in human sperm (30), local Ca^2+^ influx mediated by CatSper was suggested as an additional regulatory mechanism for hKSper activation (22).

Here, by employing fluorescent detection and current-clamp recordings, we quantitatively addressed how much physiological pH gradients and CatSper-mediated Ca^2+^ entry contribute to hKSper activation. Considering that sperm are initially stored in the epididymis at pH 5.5 to 6.8 (31, 32), subsequently exposed to the vaginal environment (pH ∼4.5), then traverse the endocervical mucus (pH ∼7.0), and ultimately reach the oviduct (pH 7.3−7.7) (33, 34, 35), we measured *V*_m_ of human sperm in response to extracellular solutions with pH 6.0, 7.0, and 8.0. In addition, as physiological hormone progesterone is known to activate CatSper (36, 37), we further evaluated the regulatory effect of progesterone on *V*_m_ hyperpolarization. Our work will help to comprehensively understand the physiological regulation of hKSper and hKSper-controlled *V*_m_ hyperpolarization during the whole fertilization process.

## Results

### Alkalization of pH_e_ in the physiological range potently activated hKSper

**Figure 1:**
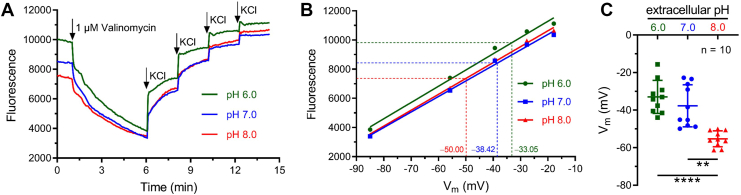
**Human sperm *V*_m_ detected by population fluorimetry was hyperpolarized in response to physiological pH_e_ increments.***A,* representative fluorescence traces showing *V*_m_ of human sperm incubated in pH_e_ 6.0, pH_e_ 7.0, or pH_e_ 8.0. Sperm *V*_m_ values corresponding to different concentrations of external K^+^ were calculated according to the Nernst equation, assuming an internal K^+^ concentration of 120 mM in human sperm (38). *Arrows* indicate time points of stimulation. *B,* calibration of fluorescence intensity and sperm *V*_m_. Resting *V*_m_ values as indicated were calculated through the equation obtained by linear regression. *C, V*_m_ values of human sperm estimated at pH_e_ 6.0, 7.0, or 8.0. *Bars* indicate the mean ± SD, n = 10 samples. Welch ANOVA tests were performed (*p* < 0.0001 and *F* = 18.78). Significant differences using Dunnett's multiple comparisons test were observed between pH_e_ 6.0 and 8.0 (*p* < 0.0001) and between pH_e_ 7.0 and 8.0 (*p* = 0.0014). *Asterisks* indicate statistical significance: *p* < 0.01 (∗∗), *p* < 0.0001 (∗∗∗∗). pH_e_, extracellular pH; *V*_m_, membrane potential.

To evaluate the contribution of physiological pH alkalization on hKSper activation, we first compared *V*_m_ of human sperm incubated with different pH_e_ by employing *V*_m_ indicator called DiSC_3_(5) (24, 38). As mentioned previously, we prepared Hepes-buffered saline (HS) solutions with pH 6.0, pH 7.0, and pH 8.0 for mimicking the physiological status of human sperm in the epididymis, uterus, and oviduct, respectively. The fluorescence signals of population *V*_m_ in human sperm preincubated at pH 6.0, 7.0, and 8.0 were respectively recorded by a multimode microplate reader (Fig. 1*A*). When the fluorescence intensity was stable, valinomycin and different volumes of KCl were injected sequentially to acquire the calibration equation between the fluorescence intensity and *V*_m_ (Fig. 1, *A* and *B*). Our statistical results showed that *V*_m_ of sperm pretreated with an acidic (pH 6.0) or a neutral (pH 7.0) solution was −32.98 ± 8.79 or −37.70 ± 11.20 mV (mean ± SD, n = 10), whereas sperm *V*_m_ significantly hyperpolarized to −55.27 ± 4.21 mV (mean ± SD, n = 10) when incubated with pH_e_ 8.0 (Fig. 1*C*).

**Figure 2:**
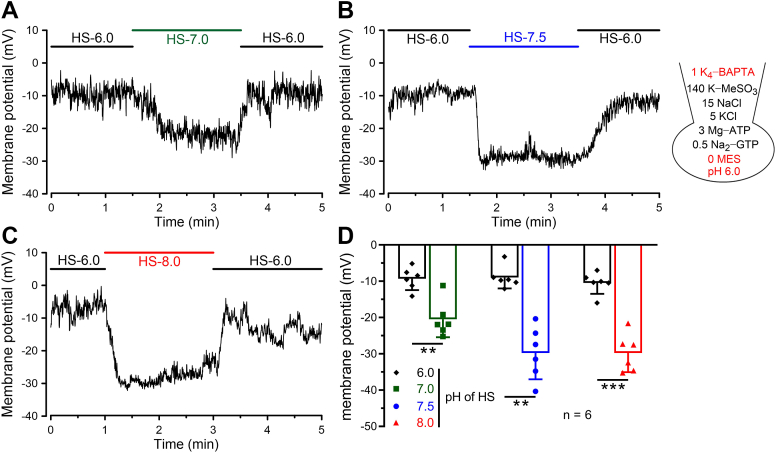
**Current-clamp recordings revealed that physiological elevations of pH_e_ caused a prominent hyperpolarization of human sperm.***A*–*C,* representative *V*_m_ traces from human sperm (pH_i_ 6.0) bathed in an acidic HS solution (pH_e_ 6.0). When recorded sperm *V*_m_ was stable, HS solution of pH 7.0 (*A*), 7.5 (*B*), or 8.0 (*C*) was perfused as indicated. The composition (in millimolar) of the pipette solution was shown in the *right panel*. *D,* mean *V*_m_ before and after the treatment of HS solutions with varying pH. *Bars* indicate the mean ± SD, n = 6 sperm cells. Significant differences using a two-tailed paired *t* test were observed between pH_e_ 6.0 and 7.0 (*p* = 0.0046), between pH_e_ 6.0 and 7.5 (*p* = 0.0015), and between pH_e_ 6.0 and 8.0 (*p* = 0.0001). *Asterisks* indicate statistical significance: *p* < 0.01 (∗∗), *p* < 0.001 (∗∗∗). HS, Hepes-buffered saline; pH_e_, extracellular pH; pH_i_, intracellular pH; *V*_m_, membrane potential.

We further detected whether *V*_m_ of a single sperm was hyperpolarized by pH alkalization in the current-clamp recordings. In the pipette solution with pH 6.0, pH buffer was removed to precisely reflect the changes of pH_i_, and the calcium chelator (1 mM 1,2-*bis*(2-aminophenoxy)-ethane-*N*,*N*,*N*′,*N*′-tetraacetic acid [BAPTA]) was added to deplete the possible alteration of *V*_m_ evoked by [Ca^2+^]_i_ changes. When the whole-cell recording mode was acquired, sperm were incubated with an acidic HS solution (pH = 6), which could ensure pH equilibrium between the inside and outside of sperm. Afterward, extracellular solutions with different pH values (7.0, 7.5, and 8.0) were applied, respectively. As shown in Figure 2, *A* and *D*, application of pH 7.0 reversibly shifted sperm *V*_m_ from −9.33 ± 3.09 mV to −20.48 ± 4.96 mV (mean ± SD, n = 6). In addition, about 20 mV hyperpolarization of *V*_m_ was observed after the application of alkalized HS solution with pH 7.5 or 8.0 (Fig. 2, *B*–*D*). Extracellular application of an NH_4_^+^ gradient has proven to be a useful way to alkalize the cell cytosol (39). As expected, high concentrations of NH_4_Cl (>5 mM) also evoked more than 15 mV hyperpolarization when sperm pH was set as 6.0 (Fig. S1). Taken together, our fluorescent and electrophysiological results supported that hKSper activity could be remarkably potentiated by physiological pH_e_ alkalization.

### Physiological Ca^2+^ entry through CatSper evoked by progesterone induced a pronounced activation of hKSper

**Figure 3:**
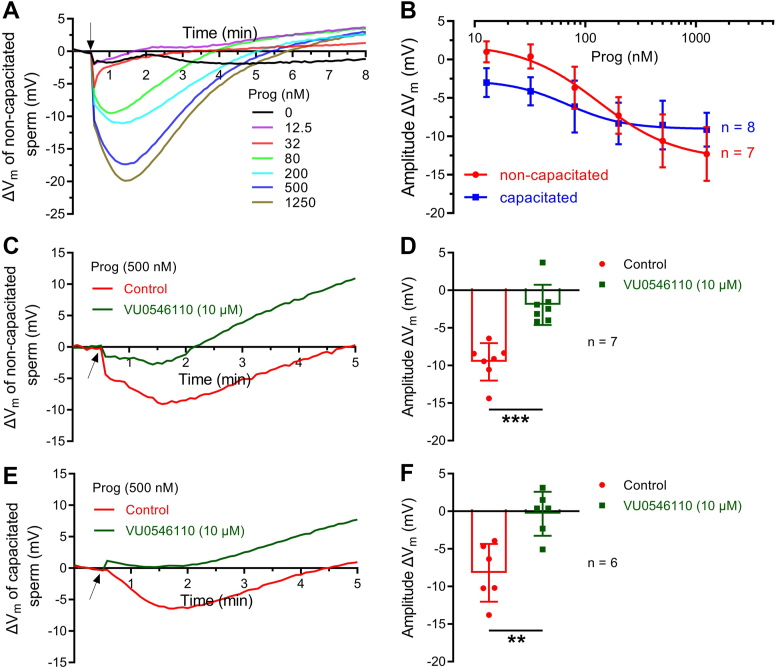
**Progesterone induced a considerable hyperpolarization of *V*_m_ through facilitating hKSper activation.***A,* representative *V*_m_ signals recorded by population fluorimetry in response to different concentrations of progesterone. *V*_m_ of noncapacitated sperm was calculated from the fluorescent intensity according to the calibration equation. *Arrows* indicate the time point at which progesterone was added. *B,* dose dependence of *V*_m_ hyperpolarized by progesterone in noncapacitated sperm (n = 7 samples) and capacitated sperm (n = 8 samples). *Bars* indicate the mean ± SD. *C* and *E,* representative progesterone-induced *V*_m_ signals of noncapacitated (*C*) or capacitated (*E*) sperm with the preincubation of 0.1% DMSO (control) or hKSper inhibitor VU0546110 (10 μM) for 30 min. *Arrows* indicate the time of progesterone stimulation. *D* and *F,* statistical analysis of the amplitude of *V*_m_ evoked by progesterone in noncapacitated (*D*, n = 7 samples) or capacitated (*F*, n = 6 samples) sperm. *Bars* indicate the mean ± SD. For (*D*), significant differences using a two-tailed unpaired *t* test with Welch's correction were observed (*p* = 0.0001). For (*F*), significant differences using a two-tailed unpaired Student's *t* test were observed (*p* = 0.0026). *Asterisks* indicate statistical significance: *p* < 0.01 (∗∗), *p* < 0.001 (∗∗∗). DMSO, dimethyl sulfoxide; hKSper, human KSper; *V*_m_, membrane potential.

As mentioned previously, progesterone-stimulated CatSper activation is the main Ca^2+^ intake pathway in human sperm (36, 37). To assess the effect of physiological Ca^2+^ signaling on hKSper, we examined the effect of progesterone at different concentrations on hKSper by quantitatively measuring population *V*_m_ of purified human sperm loaded with DiSC_3_(5). The concentration of progesterone utilized was up to 1.25 μM, since progesterone at higher concentrations might be out of physiological significance and inhibit hKSper current (22, 23, 40). Our results showed that progesterone evoked a transient and dramatic hyperpolarization followed by a weak depolarization (Fig. 3*A*). The dose–response relationship of progesterone on sperm hyperpolarization revealed that the EC_50_ was 131.88 ± 101.11 nM (mean ± SD, n = 7) (Fig. 3*B*). It was noted that during the fertilization process, sperm need to be capacitated in the oviductal reservoir and subsequently released to the fallopian tube containing a high concentration of progesterone (41). Therefore, we also established the dose–response curve of progesterone on capacitated sperm hyperpolarization (Fig. 3*B*). By comparison with noncapacitated sperm, the dose–response curve for capacitated sperm was less steep, and its EC_50_ of progesterone was relatively lower (69.38 ± 108.20 nM, mean ± SD, n = 8) (Fig. 3*B*).

Next, to clarify whether progesterone-stimulated hyperpolarization of *V*_m_ is mediated by hKSper, clofilium and VU0546110, the pharmacological inhibitors for hKSper, were employed in population *V*_m_ detection (22, 24). Although clofilium partially impaired progesterone-generated Ca^2+^ influx (Fig. S2, *A* and *B*), probably owing to the inhibition of CatSper (28), it completely eliminated the hyperpolarization of *V*_m_ evoked by progesterone (Fig. S2, *C* and *D*). In the case of VU0546110, whether it regulates CatSper activity has not been determined. Here, we found that VU0546110 had no impact on progesterone-induced Ca^2+^ influx (Fig. S3), and sperm hyperpolarization evoked by progesterone was almost suppressed after incubating with VU0546110 on both noncapacitated (Fig. S3, *C* and *D*) and capacitated sperm (Fig. S3, *E* and *F*). Taken together, our results supported that hKSper was responsible for this physiologically relevant *V*_m_ hyperpolarization caused by progesterone.

**Figure 4:**
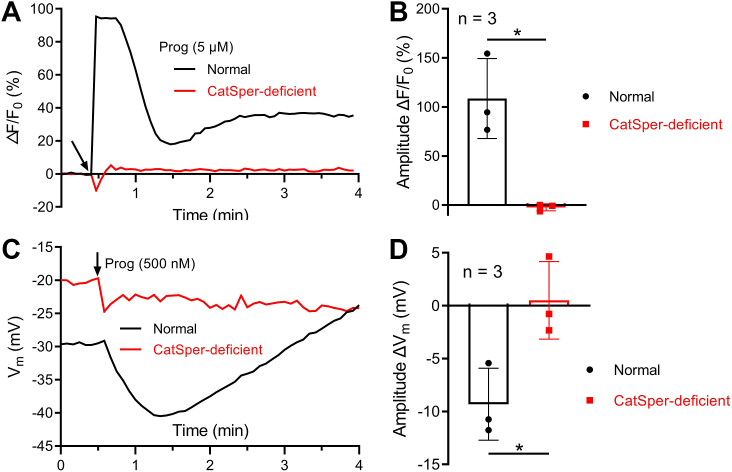
**CatSper activity was required for progesterone-induced hyperpolarization of *V*_m_.***A* and *C,* representative fluorescent signals of Ca^2+^ (*A*) or *V*_m_ (*C*) evoked by progesterone in a normal or CatSper-deficient sample. Human sperm *V*_m_ was calculated from the fluorescent intensity according to the calibration equation. *Arrows* indicate the time of stimulation. *B* and *D,* statistical analysis of the amplitude of progesterone-induced Ca^2+^ (*B*) or *V*_m_ (*D*) signals of human sperm from different normal samples (n = 3 biological replicates) and from the CatSper-deficient sample (n = 3 technique replicates). *Bars* indicate the mean ± SD. For the comparison of Ca^2+^ signals between normal and CatSper-deficient samples (*B*), significant differences using a two-tailed unpaired *t* test with Welch's correction were observed (*p* = 0.0412). For the comparison of *V*_m_ signals between normal and CatSper-deficient samples (*D*), significant differences using a two-tailed unpaired Student's *t* test were observed (*p* = 0.0272). *Asterisks* indicate statistical significance: *p* < 0.05 (∗). CatSper, sperm-specific Ca2+ channel; *V*_m_, membrane potential.

Subsequently, whether CatSper-mediated Ca^2+^ signaling participated in the regulatory function of progesterone on hKSper was explored. We did not choose two potent CatSper inhibitors, NNC 55-0396 and mibefradil, owing to their possible off-target effects on hKSper inhibition (28). Fortunately, we recruited an infertile patient whose sperm lack the monovalent ion currents mediated by CatSper (Fig. S4). In addition, the hKSper currents recorded from this CatSper-deficient sample were comparable to that recorded from normal sperm (Fig. S5). Notably, the sample collected from this patient was also utilized in a previous study (42). The undetectable Ca^2+^ response of progesterone on this specific sample verified that this patient exhibited a functional deficiency of CatSper (Fig. 4, *A* and *B*). Fluorescent *V*_m_ detection showed that progesterone failed to produce any noticeable hyperpolarization of *V*_m_ in this CatSper-deficient sample (Fig. 4, *C* and *D*). Therefore, these results indicated that CatSper activity was essential for progesterone-evoked hKSper activation.

By employing the high-speed fluorescent recordings, we tried to further scrutinize the temporal sequence of hKSper and CatSper activation in response to progesterone. However, because of the limitation of the experimental device, the initial fluorescent signals (within ∼7 s) after injecting the stimuli into the recording plates could not be documented. Nevertheless, as shown in Figure S6*A*, we observed distinct kinetic profiles for Ca^2+^ and *V*_m_ signaling following progesterone stimulation. The statistical analysis showed that there was about 30 s' latency between the peak time point of Ca^2+^ and *V*_m_ signaling (Fig. S6*B*), possibly reflecting the gating kinetic differences between CatSper and hKSper channels and/or the time required for Ca^2+^ diffusion to hKSper.

In summary, our fluorometric results convincingly demonstrated that progesterone at the physiological concentration activated hKSper and evoked about 15 mV hyperpolarization of *V*_m_ through CatSper-mediated Ca^2+^ signals.

### Current-clamp recordings revealed a regulatory effect of resting [Ca^2+^]_i_ level on hKSper activation

**Figure 5:**
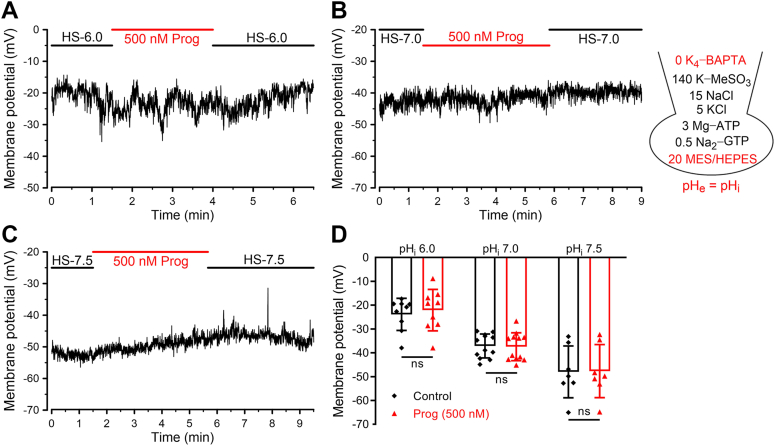
**Progesterone failed to hyperpolarize *V*_m_ at varying pH_i_ in the absence of intracellular BAPTA.***A*–*C,* representative *V*_m_ traces recorded by current clamp at pH_i_ 6.0 (*A*), 7.0 (*B*), and 7.5 (*C*). The composition (in millimolar) of the pipette solution was shown in the *right panel*. The pH values of pipette solutions were in accordance with pH_e_. *D,* the mean *V*_m_ before and after the perfusion of progesterone (500 nM) at the indicated pH_i_. *Bars* indicate the mean ± SD, n = 7 to 12 sperm cells. Exact *p* values using a two-tailed paired *t* test were pH_i_ 6.0, *p* = 0.2551; pH_i_ 7.0, *p* = 0.7288; pH_i_ 8.0, *p* = 0.7656. ns, not significant; *p* ≥ 0.05. BAPTA, 1,2-*bis*(2-aminophenoxy)-ethane-*N*,*N*,*N*′,*N*′-tetraacetic acid; pH_e_, extracellular pH; pH_i_, intracellular pH; *V*_m_, membrane potential.

Next, we tried to confirm the effect of progesterone on hKSper by utilizing the current-clamp recordings. For the pipette solution, Ca^2+^ chelator (1 mM BAPTA) was removed to permit the elevation of [Ca^2+^]_i_. Given that progesterone-evoked Ca^2+^ entry through CatSper in human sperm is pH sensitive (37), three kinds of pipette solutions with pH 6.0, 7.0, and 7.5 were prepared to evaluate the potential effect of different amplitudes of Ca^2+^ influx on hKSper. The pH buffer (20 mM Mes or Hepes) was supplemented according to the pipette pH, although progesterone did not change sperm pH_i_ (36). Surprisingly, as shown in Figure 5, *A* and *D*, there was no significant hyperpolarization of *V*_m_ at pH_i_ 6.0 by the treatment of 0.5 μM progesterone. After alkalizing the pH of the pipette solution to 7.0 or 7.5, progesterone still failed to induce the hyperpolarization of *V*_m_ (Fig. 5, *B*–*D*), although resting *V*_m_ was hyperpolarized from −23.87 ± 2.12 mV (pH_i_ 6.0, mean ± SD, n = 9) to −37.13 ± 1.38 mV (pH_i_ 7.0, mean ± SD, n = 12) or −48.0 ± 3.81 mV (pH_i_ 7.5, mean ± SD, n = 7) (Fig. 5, *B*–*D*), which was consistent with our aforementioned results with regard to the physiological activation of hKSper evoked by pH_e_ alkalization (Fig. 2).

**Figure 6:**
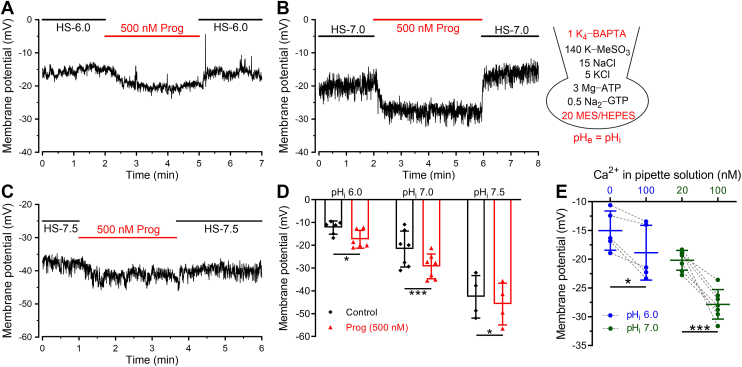
**Progesterone caused the hyperpolarization of *V*_m_ at different pH_i_ in the presence of intracellular BAPTA.***A*–*C,* representative *V*_m_ traces recorded by current clamp at pH_i_ 6.0 (*A*), 7.0 (*B*), and 7.5 (*C*). The composition (in millimolar) of the pipette solution was shown in the *right panel*, and its pH was in accordance with pH_e_. *D,* mean *V*_m_ before and after the perfusion of progesterone (500 nM) at pH_i_ 6.0 (*A*, n = 7 sperm cells), pH_i_ 7.0 (*B*, n = 7 sperm cells), and pH_i_ 8.0 (*C*, n = 4 sperm cells). *Bars* indicate the mean ± SD. Exact *p* values using a two-tailed paired *t* test were pH_i_ 6.0, *p* = 0.0120; pH_i_ 7.0, *p* = 0.0006; and pH_i_ 8.0, *p* = 0.0305. *E,* mean *V*_m_ recorded from human sperm in the absence or presence of basal [Ca^2+^]_i_ (100 nM) when sperm pH_i_ was set to 6.0 (*left panel*, n = 5 sperm cells) or 7.0 (*right panel*, n = 7 sperm cells). The *dashed lines* indicate that *V*_m_ was recorded from the same sample. *Bars* indicate the mean ± SD. Exact *p* values using a two-tailed paired *t* test were pH_i_ 6.0, *p* = 0.0172; pH_i_ 7.0, *p* = 0.0003. *Asterisks* indicate statistical significance: *p* < 0.05 (∗), *p* < 0.001 (∗∗∗). BAPTA, 1,2-*bis*(2-aminophenoxy)-ethane-*N*,*N*,*N*′,*N*′-tetraacetic acid; [Ca^2+^]_i_, intracellular Ca^2+^; pH_e_, extracellular pH; pH_i_, intracellular pH; *V*_m_, membrane potential.

It was suggested that without Ca^2+^ chelator, free Ca^2+^ at micromolar ranges contained in the distilled water would give rise to contaminant Ca^2+^ in the pipette solution (22). We speculated that in the whole-cell configuration, this contaminant of Ca^2+^ would enter into sperm cytoplasm and evoke the sufficient activation of hKSper, which could prevent progesterone from further activating hKSper. Indeed, compared with the BAPTA-free condition, adding BAPTA to the pipette solution to chelate contaminant Ca^2+^ could cause *V*_m_ depolarization, suggesting that there was less hKSper activation when contaminant Ca^2+^ was ablated (Fig. S7). Thus, to minimize the disturbing effect of residual Ca^2+^ of the pipette solution on hKSper, 1 mM BAPTA was added in the pipette solution. Interestingly, we observed a significant hyperpolarization of *V*_m_ induced by progesterone at pH_i_ 6.0 (from −12.24 ± 2.91 mV to −17.39 ± 3.90 mV, mean ± SD, n = 6, Fig. 6*A*) and at pH_i_ 7.0 (from −21.65 ± 7.89 mV to −29.26 ± 5.42 mV, mean ± SD, n = 7, Fig. 6*B*). Considering that progesterone-caused Ca^2+^ influx might be partially compromised by the Ca^2+^ chelation effect of BAPTA, these current-clamping recording results suggested that progesterone caused at least 8 mV of *V*_m_ hyperpolarization on human sperm, generally consistent with our population fluorescence measurements (10−15 mV, Fig. 3). Whereas, a relatively weak effect of progesterone on hKSper was shown at pH_i_ 7.5 (from −42.59 ± 9.32 mV to −45.77 ± 9.13 mV, mean ± SD, n = 4), which possibly resulted from the saturated activation of hKSper induced by pH_i_ alkalization (Fig. 6, *C* and *D*).

To further clarify the regulatory role of basal [Ca^2+^]_i_ signaling on hKSper, we assessed whether the alteration within a range of nanomolar [Ca^2+^]_i_ concentration had an impact on hKSper activity. To avoid the potential difference between sperm samples, we detected that *V*_m_ of human sperm comes from the same sample by employing the recording conditions with different [Ca^2+^]_i_. Our results showed that compared with *V*_m_ of human sperm containing nominal [Ca^2+^]_i_, the elevation of [Ca^2+^]_i_ to 100 nM induced a hyperpolarization of *V*_m_ (Fig. 6*E*). In addition, when pH_i_ was elevated to 7.0, the hyperpolarization of *V*_m_ induced by increasing [Ca^2+^]_i_ from 20 nM to 100 nM became more dramatical (Fig. 6*E*), hinting that pH alkalization was capable of enhancing the activating effect of basal [Ca^2+^]_i_ on hKSper. Taken together, our findings supported that the maintenance of resting [Ca^2+^]_i_ level in human sperm contributed to hKSper activity.

## Discussion

The hyperpolarization of sperm *V*_m_ was demonstrated to be positively correlated with its fertilizing capacity (8, 9). hKSper, a K^+^ channel mainly determining *V*_m_ of sperm, plays an important role in the modulation of sperm functions (10). However, the physiological regulation of native hKSper was far from elucidated. Here, by performing quantitative *V*_m_ measurements and electrophysiological recordings, we managed to elucidate the crucial role of physiological pH_e_ elevation and CatSper-mediated Ca^2+^ signaling for enhancing the activation of the hKSper channel.

### Physiological pH_e_ alkalization enhanced hKSper activation

Our results found that pH_e_ alkalization that occurs during the fertilization process was capable of potentiating hKSper and inducing at least 20 mV hyperpolarization of *V*_m_, either by population fluorescence measurement (Fig. 1) or by current-clamp recording (Fig. 2), which emphasized that hKSper could be activated and participate in regulating sperm function by sensing the physiological pH stimuli. A previous electrophysiological report has shown that hKSper was insensitive to intracellular alkalization by employing the pipette solution containing 5 mM Hepes (23). In contrast, in this study, hKSper activation elicited by pH_e_ alkalization or NH_4_Cl could be detected with pH buffer–free pipette solution (Figs. 2 and S1). Besides, considering that under the physiological conditions, pH alkalization is able to evoke CatSper-mediated Ca^2+^ elevation, which would potentiate hKSper (36, 37, Fig. 3), the Ca^2+^ chelator (1 mM BAPTA) was added in the pipette solution of our current-clamping experiments when evaluating pH_e_-caused hKSper activation (Figs. 2 and S1). However, Ca^2+^-dependent activation of hKSper may have somewhat contributed to the measurements when utilizing the population fluorescence method (Fig. 1). Nevertheless, we demonstrated a sufficient activation of hKSper induced by physiological pH_e_ alkalization.

### CatSper-mediated Ca^2+^ influx could activate hKSper

The increase of [Ca^2+^]_i_ >10 μM was reported to dramatically potentiate the hKSper channel (22, 23), although the molecular mechanism of this Ca^2+^-sensitive property has not yet been solved. In our results, by utilizing population *V*_m_ measurements, we first clarified that progesterone at the physiological concentration evoked 10 to 15 mV hyperpolarization of *V*_m_ (Fig. 3). Notably, a previous report has showed that progesterone at 10 μM transiently depolarized *V*_m_ followed by a repolarization phase (43). We reasoned that the inhibitory effect of progesterone (10 μM) on hKSper underlay this initial depolarization, and subsequently, progesterone-induced Ca^2+^ influx through CatSper brought about the rapid hyperpolarization of *V*_m_ (22, 23, 44).

In addition, we observed the changes in both the EC_50_ and slope of progesterone-caused *V*_m_ hyperpolarization during capacitation (Fig. 3*B*). In fact, a previous report showed that the EC_50_ of progesterone for eliciting Ca^2+^ signals shifted significantly during capacitation—from 42 ± 15 nM in noncapacitated sperm to 10 ± 10 nM in capacitated sperm (36). This shift likely underlies the corresponding changes in the EC_50_ of progesterone-induced *V*_m_ hyperpolarization observed in our study (from 131.88 ± 101.11 nM to 69.38 ± 108.20 nM) (Fig. 3*B*), as progesterone-mediated *V*_m_ hyperpolarization is driven by CatSper-dependent Ca^2+^ influx (Fig. 4). How to explain why progesterone at lower concentrations caused more *V*_m_ hyperpolarization but less *V*_m_ hyperpolarization at higher concentrations during capacitation compared with the noncapacitation condition (Fig. 3*B*)? As a fact, capacitation elevates pH_i_ (45), which enhances the activities of both CatSper and KSper (22, 36, 37). We speculate that, in capacitated sperm, because more CatSper was activated, progesterone at lower concentrations might trigger greater Ca^2+^ entry and then more pronounced *V*_m_ hyperpolarization. However, since hKSper was also more activated and thus the intrinsic *V*_m_ was more hyperpolarized in capacitated sperm, the Ca^2+^-dependent activation of hKSper caused by higher concentrations of progesterone would be compromised since the hKSper activation reached a “saturation” level. On the other hand, the possible interaction between pH and Ca^2+^-dependent regulation of hKSper may also contribute to the slope difference between noncapacitated and capacitated sperm observed here, which definitely needs further investigation.

By employing the specific inhibitor of SLO3 (VU0546110) and an infertile patient suffering from the loss of functional CatSper, we confirmed that progesterone-induced *V*_m_ hyperpolarization arose from CatSper-mediated hKSper activation (Figs. 3, *C*–*F* and 4). In addition, different from other SLO3 inhibitors, such as clofilium and quinidine, which suppress CatSper activity (28), VU0546110 had a marginal effect on CatSper-mediated Ca^2+^ entry (Fig. S3), which suggests that the inhibitory function of VU0546110 on acrosome reaction and hyperactivation previously reported probably resulted from the reduction of hKSper activity (24).

### What is the physiological significance of hKSper activation evoked by Ca^2+^ influx?

In distinction from the sustained hyperpolarization of *V*_m_ evoked by pH_i_ alkalization (Figs. 1 and 2), our results showed that progesterone initially augmented the open probability of hKSper during the transient elevation of [Ca^2+^]_i_ (Figs. 3*A* and 4*A*). Afterward, the activation of hKSper was gradually reduced (Fig. 3*A*). It has been well elucidated that the instantaneous Ca^2+^ signals of human sperm caused by progesterone promoted a highly asymmetrical pattern of flagellar wave, which is a hallmark of hyperactivity (46). But what is the physiological significance of the transient activation of hKSper? Since CatSper is a voltage-dependent channel essential for sperm-fertilizing capacity (30), the physiological significance of Ca^2+^ dependence of hKSper is suggested to regulate CatSper. On one hand, hKSper activation caused *V*_m_ hyperpolarization, which may function as the negative feedback to attenuate CatSper activation (22). This process could potently hamper excessive Ca^2+^ influx. On the other hand, the hyperpolarization of *V*_m_ through Ca^2+^-activated hKSper would enhance the electrical driving force of Ca^2+^ and further facilitate Ca^2+^ influx through CatSper (47). Alternatively, hKSper may play physiological roles through modulating other *V*_m_-dependent molecules (48). Interestingly, our recent work found that the depolarization of *V*_m_ evoked by hKSper inhibition decreased pH_i,_ probably through suppressing the activities of voltage-sensitive Na^+^/H^+^ exchangers (49). Thus, given the pH sensitivity of CatSper, hKSper may indirectly affect the activities of CatSper through *V*_m_-sensitive pH in human sperm. Considering the distinct ways for hKSper to regulate CatSper, the net effect of hKSper on CatSper activation perhaps relies on the experimental conditions. Nevertheless, our previous study suggested that inhibition of the hKSper subunit LRRC52 could attenuate Ca^2+^ signaling in human sperm (27). In addition, quinidine as the potent inhibitor for hKSper could arrest [Ca^2+^]_i_ oscillation in human sperm (50). These reports suggest that the general effect of hKSper is to potentiate CatSper activation. However, in this study, both the progesterone-elicited [Ca^2+^]_i_ increase and the decreasing slopes of progesterone-induced Ca^2+^ signals in the absence or presence of the hKSper inhibitor VU0546110 were substantially comparable (Fig. S3), raising concern regarding the net effect of hKSper on CatSper regulation. Taking advantage of the human sperm samples with the defects of SLO3 function (25, 26) and/or completely evaluating the specificity of the hKSper inhibitors over CatSper by electrophysiological methods should help to clarify this discrepancy.

Interestingly, by comparison with population [Ca^2+^]_i_ measurements, previous studies revealed by single-cell [Ca^2+^]_i_ imaging on human sperm that when responding to progesterone, sperm exhibited different [Ca^2+^]_i_ patterns, including oscillatory [Ca^2+^]_i_ signal, transitory [Ca^2+^]_i_ burst, and sustained [Ca^2+^]_i_ elevation (51). Based on our results, we speculate that there should be different patterns of Ca^2+^-activated hKSper activation in the human sperm with specific [Ca^2+^]_i_ signals. In fact, the hyperpolarization of *V*_m_ caused by the K^+^ ionophore termed valinomycin robustly suppressed [Ca^2+^]_i_ oscillation in human sperm, suggesting a potential role of hKSper on [Ca^2+^]_i_ oscillation (50). The employment of VU0546110 on single-sperm [Ca^2+^]_i_ imaging will be beneficial for better elucidating the involvement of hKSper in the regulation of [Ca^2+^]_i_ oscillation. Nevertheless, given a potential role of [Ca^2+^]_i_ oscillation on sperm fertility (52), we imply that reciprocal regulation of Ca^2+^ signaling and hKSper activity should modulate sperm functions.

### hSLO3-mediated hKSper may harbor two Ca^2+^-binding sites with low and high affinities

Based on our electrophysiological results, we demonstrated that resting [Ca^2+^]_i_ around 100 nM could regulate hKSper activity and cause about 10 mV *V*_m_ hyperpolarization (Fig. 6*E*). In fact, a previous study has reported the hKSper potentiation by similar [Ca^2+^]_i_ (23). Notably, the resting *V*_m_ of human sperm collected from the CatSper-deficient patient was relatively depolarized compared with that from normal sperm (Fig. 4*C*), perhaps reflecting the reduction of basal [Ca^2+^]_i_ resulting from the functional abnormality of CatSper (53), which in turn downregulated hKSper activation. All the aforementioned information suggests that hKSper contains a high-affinity Ca^2+^-binding site. Combined with the report that [Ca^2+^]_i_ elevation above 50 μM facilitated hKSper activation (22, 23), we propose that hKSper may harbor dual Ca^2+^ regulatory sites with high and low sensitivity.

The ability of hKSper to sense low and high concentrations of Ca^2+^ reminisces the Ca^2+^ sensitivity of human SLO1 (hSLO1) (54). In fact, there has been a long-term controversy about whether hKSper is conducted by hSLO3 or hSLO1. Recent advances in SLO3-specific pharmacology, particularly the development of the selective inhibitor VU0546110, have established hSLO3 rather than hSLO1 as the sole K^+^ channel responsible for the hyperpolarization of *V*_m_ in human sperm (24). Moreover, unlike mSLO3, heterogeneous expression proved that hSLO3 exhibited dual sensitivity to both pH_i_ and Ca^2+^ (22), supporting that hSLO3 alone can account for the dual regulatory mechanism of hKSper. In this study, by employing VU0546110, we demonstrated that hSLO3 was fully responsible for the progesterone-elicited Ca^2+^-dependent *V*_m_ hyperpolarization (Fig. 3, *C*–*F*). The aforementioned findings all support that hSLO3 is the pore-forming subunit of hKSper. Regarding the mechanisms by which hKSper/hSLO3 senses Ca^2+^, by comparing the amino acid sequence between hSLO1 and hLSO3, several negatively charged amino acid residues essential for Ca^2+^ binding in hSLO1 could not be detected in hSLO3 (55). Additionally, whether the auxiliary LRRC52 contributes to Ca^2+^ sensitivity of hKSper might be worthy of investigation, considering that LRRC52 robustly amplified hSLO3 currents in a co-expression system (56). So far, the exact molecular mechanism of the Ca^2+^ dependence of hKSper/hSLO3 activation still remains obscure (10). Unveiling the conformational transition of the hSLO3–LRRC52 complex in the presence of different Ca^2+^ accumulations or pH alkalizations will contribute to pinpointing this unsolved question.

In conclusion, our work that focuses on the physiological activation of hKSper benefits for better understanding the fundamental mechanism of hKSper regulation and, moreover, strengthens the significance of hKSper activation on sperm function and male fertility.

## Experimental procedures

### Reagents

DiSC_3_(5) was obtained from AAT Bioquest. Commercial human tubal fluid medium containing bovine serum albumin (4 mg/ml) and NaHCO_3_ (25 mM) was acquired from Aibei Biotechnology. Fluo-4 AM and F-127 were obtained from Invitrogen (ThermoFisher Scientific). Valinomycin and clofilium were obtained from MCE (MedChemExpress). VU0546110 (product ID: F6374-1749) was purchased from Life Chemicals. Progesterone and other compounds were acquired from Sigma–Aldrich, if not stated otherwise.

### Sperm preparation

The studies on human sperm were performed in agreement with the requirements set by the Declaration of Helsinki principles. The collections and experiments of human sperm samples were approved by the Institutional Ethics Committee of the affiliate hospital of Nantong University. Freshly ejaculated sperm samples were obtained by masturbation from healthy volunteers with their prior consent. Sperm semen was allowed to liquefy for 30 to 60 min at room temperature. For the electrophysiological recordings, sperm were centrifuged and resuspended with HS solution (in millimolar): 135 NaCl, 5 KCl, 1 MgSO_4_, 2 CaCl_2_, 5 glucose, 10 lactic acid, 1 sodium pyruvate, and 20 Hepes adjusted to pH 7.4 with NaOH. The washing step was performed two or three times to completely remove it from the seminal fluid. For the measurements of [Ca^2+^]_i_ and *V*_m_, sperm semen was purified by swim-up in HS solution at 37 °C for 1 h. Motile sperm were centrifuged for 6 min at 300*g* and capacitated in commercial human tubal fluid medium containing 25 mM NaHCO_3_ and 4 mg/ml bovine serum albumin at 37 °C for 4 h.

### Measurement of changes in [Ca^2+^]_i_ and *V*_m_

The measurements of [Ca^2+^]_i_ and *V*_m_ in human sperm were detected by the Synergy HTX Multi-Mode Reader (Biotek Instruments). For the determination of *V*_m_ in response to pH_e_ alkalization, purified sperm (8 × 10^6^ ml^–1^) were equally divided and pretreated into HS solution with pH 6.0, 7.0, and 8.0 for 30 min. Particularly, the pH buffer (20 mM Hepes) was substituted for Mes (20 mM) in HS solution with pH 6.0. After incubation, sperm were loaded with 2 μM DiSC_3_(5) for 10 min at 37 °C in the dark. Subsequently, 90 μl sperm suspensions were added in a 96-well plate. The fluorescence was excited at 640 nm, and the resulting emission was detected at 680 nm. For the measurements of *V*_m_ at different pH_e_ solutions, 10 μl valinomycin (10 μM, 1:10 dilution) followed by 0.5 μl, 0.65 μl, 0.8 μl, and 1.05 μl KCl (2 M) were added sequentially. Assuming that the internal K^+^ concentration is 120 mM in human sperm (57), the corresponding *V*_m_ was −84.93 mV, −55.78 mV, −39.24 mV, −27.29 mV, and −17.12 mV according to the Nernst equation. Every sperm suspension in a well has a unique calibration equation. For the measurement of *V*_m_ in response to progesterone, 10 μl of chemicals were injected into sperm suspensions (1:10 dilution). An extra well was used to acquire the calibration equation. Every sperm sample has a unique calibration equation. All *R*^2^ values calculated by linear regression were >98%. EC_50_ was calculated by GraphPad Prism 9 (GraphPad Software).

For the determination of [Ca^2+^]_i_ changes, purified sperm (2 × 10^7^ ml^–1^) were loaded with 5 μM Fluo-4 AM supplemented with 0.05% pluronic F-127 for 30 to 40 min at 37 °C in the dark (36). After incubation, excess dye was removed by centrifugation. Sperm suspensions (90 μl) were filled in a 96-well plate. The fluorescence was excited at 488 nm, and the fluorescence emission was recorded at 528 nm. When the fluorescent intensity was stable, specific chemicals were injected into sperm suspensions (1:10 dilution). Changes of [Ca^2+^]_i_ were depicted as ΔF/F_0_.

For high-speed recordings of *V*_m_ and [Ca^2+^]_i_ signals, purified human sperm were equally divided and loaded with DiSC_3_(5) and Fluo-4 AM, respectively. Sperm suspensions were incubated in an individual well, followed by progesterone stimulation. The interval time for the acquisition of fluorescent signals was set to 2 ms. The subsequent procedures were carried out as described previously.

### Patch-clamp recordings of human sperm

The whole-cell patch-clamp recordings on human sperm were performed by employing Axon 200B (Molecular Devices) as described previously (22, 58). Briefly, human sperm were washed three times to remove the seminal fluid, after which the sperm heads were allowed to settle and adhere to a 35-mm cell culture dish (Corning Incorporated). Seals were formed at the cytoplasmic droplet of the neck region in sperm cells by a 20 to 35 MΩ pipette. The whole-cell configuration was achieved by a zap protocol (400–650 mV, 0.5 ms) combined with synchronous suction. The current clamp mode was switched for the detection of *V*_m_ of human sperm under the specific conditions. To test the pH sensitivity of hKSper, the HS solution was adjusted to pH 6.0, 7.0, 7.5, or 8.0 by NaOH. The pipette solution contains (in millimolar): 140 K–MeSO_3_, 5 KCl, 15 NaCl, 3 Mg–ATP, 0.5 Na_2_–GTP, 1 BAPTA adjusted to pH 6.0 with KOH. The pipette solution with 100 nM Ca^2+^ contains (in millimolar): 140 K–MeSO_3_, 2.5 CaCl_2_, 15 NaCl, 5 EGTA, 20 Hepes, 3 Mg–ATP, 0.5 Na_2_–GTP adjusted to pH 7.3 with KOH. To test Ca^2+^ sensitivity of hKSper, the pipette solution with or without Ca^2+^ chelator contains (in millimolar): 140 K–MeSO_3_, 5 KCl, 15 NaCl, 20 Mes/Hepes, 0/1 BAPTA, 3 Mg–ATP, 0.5 Na_2_–GTP, adjusted to pH 6.0, 7.0, or 7.5 with KOH. The pipette solution with 0 or 100 nM Ca^2+^ contains (in millimolar): 140 K–MeSO_3_, 4.76 KCl, 0/0.12 CaCl_2_, 15 NaCl, 3 Mg–ATP, 0.5 Na_2_–GTP, 1 BAPTA, and 20 Mes adjusted to pH 6.0 with KOH. The pipette solution with 20 or 100 nM Ca^2+^ contains (in millimolar): 140 K–MeSO_3_, 4.44/3.4 KCl, 0.28/0.8 CaCl_2_, 15 NaCl, 1 EDTA, 1 EGTA, 3 Mg–ATP, 0.5 Na_2_–GTP, 1 BAPTA, and 20 Hepes adjusted to pH 7.0 with KOH. Desired free Ca^2+^ concentrations were calculated by WINMAXC32, version 2.51 (Chris Patton, Stanford University). Mes instead of Hepes was chosen as the pH buffer when the pH of the solution was 6.0. The osmolarity of the extracellular or intracellular solutions was 310 to 330 mOsm. The extracellular solutions were injected using a homemade perfusion system. Liquid junction potential was corrected during *V*_m_ recording. Data were analyzed with Clampfit 10.4 (Molecular Devices) and Grapher 15 (Golden Software).

### Statistical analysis

Data are expressed as mean ± SD. The normal probability plot and variance homogeneity test were employed to assess the normality of data distributions and the homogeneity of variance, respectively. To determine significant differences indicative of changes among the groups with GraphPad Prism 9 (GraphPad Software), *t* test for two groups and ANOVA followed by appropriate *post hoc* tests for multiple groups were used. The detailed statistical test methodologies and exact *p* values have been described in the corresponding legends of figures. Differences with a *p* < 0.05 were considered statistically significant.

## Data availability

The data underlying this article will be shared on reasonable request to the corresponding author.

## Supporting information

This article contains supporting information.

## Conflict of interest

The authors declare that they have no conflicts of interest with the contents of this article.
