# Supplementary material for: Human sperm KSper is physiologically activated by intracellular pH alkalization and CatSper-mediated Ca2+ signaling
Source: J Biol Chem. 2025 Sep 22;301(11):110752. doi: 10.1016/j.jbc.2025.110752 (PMC12569824; doi:10.1016/j.jbc.2025.110752)
Supplement: Supplementary Figures [file mmc1.docx]

Human sperm KSper is physiologically activated by intracellular pH alkalization and CatSper-mediated Ca^2+^ signaling

Hang Kang^1,‡^, Huafeng Wang^2,‡,§^, Jie Wu^1^, Jiali Zhang^1^, Xiaoning Zhang^1,^*, Xuhui Zeng^1,^*

^1^Institute of Reproductive Medicine, Medical school, Nantong University, Nantong, Jiangsu, 226019, China

^2^Institute of Life Science and School of Life Science, Nanchang University, Nanchang, Jiangxi, 330031, China

^‡^These authors contributed equally to this work.

^§^Present address: Department of Pharmacology, Yale University School of Medicine, New Haven, CT, 06510.

*Corresponding address: Zhang Xiaoning, zhangxn@ntu.edu.cn; Zeng Xuhui, zengxuhui@ntu.edu.cn.


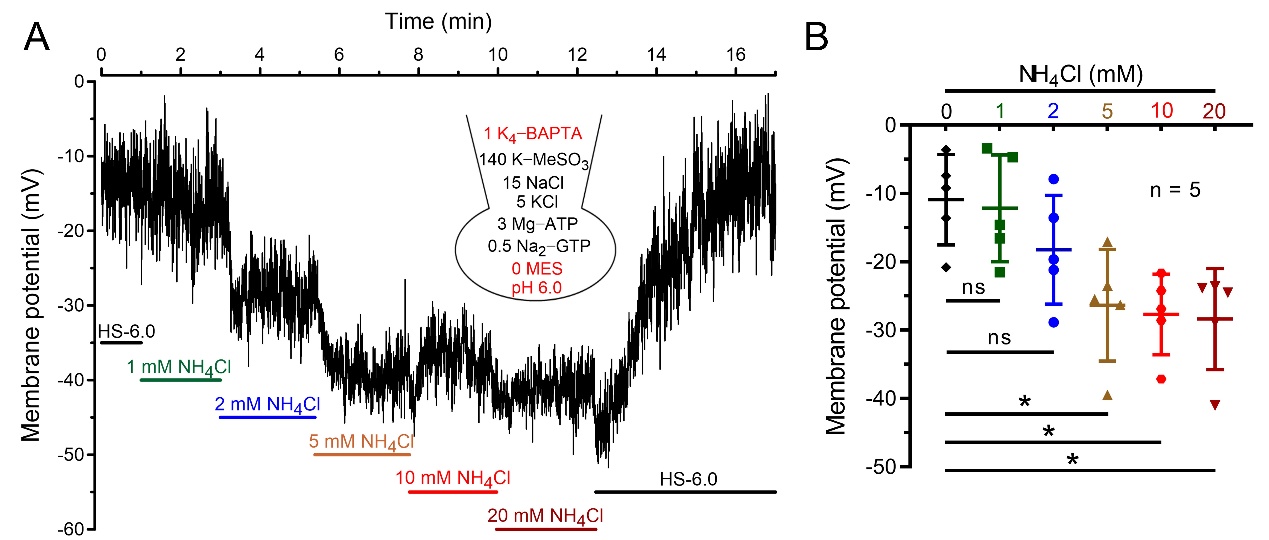


**Supplementary Figure 1. Current-clamp recordings revealed an alkalization-activated property of hKSper.** (**A**) Representative V_m_ recordings from human sperm (pH_i_ 6.0) bathed in an acidic HS solution (pH 6.0) in response to NH_4_Cl-evoked intracellular alkalization were shown. The pH buffer was removed from the pipette solution. (**B**) Mean V_m_ under conditions as described in **A**. Bars indicate the mean ± SD, n = 5 sperm cells. Repeated measures one-way ANOVA tests were performed (*p* = 0.0018 and F = 20.29). Exact *p* values using Dunnett’s multiple comparisons test were: 1 mM NH_4_Cl, *p* = 0.8766; 2 mM NH_4_Cl, *p* = 0.2449; 5 mM NH_4_Cl, *p* = 0.0453; 10 mM NH_4_Cl, *p* = 0.0114; 20 mM NH_4_Cl, *p* = 0.0174 versus control. Asterisks indicate statistical significance: *p* < 0.05 (*). ns not significant, *p* ≥ 0.05.


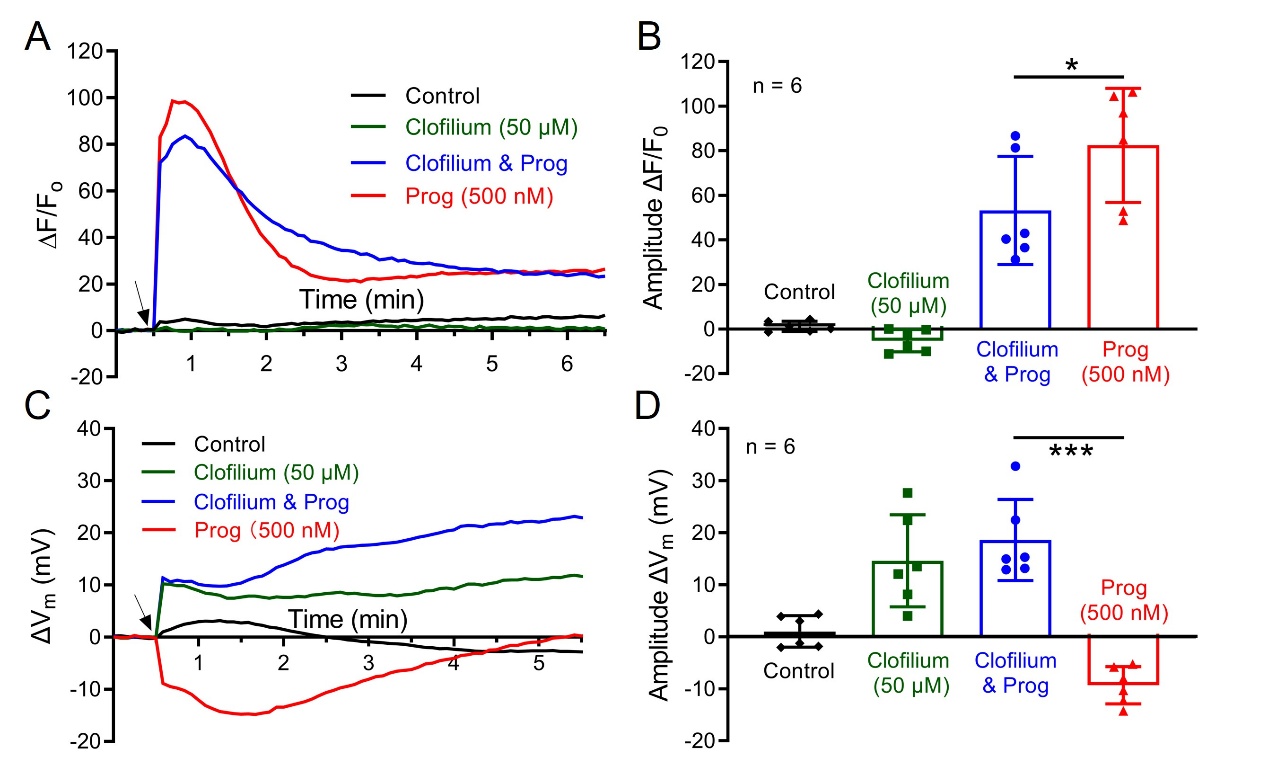


**Supplementary Figure 2. The inhibitory effect of clofilium on progesterone-induced Ca^2+^ signals and V_m_ hyperpolarization**. (**A, C**) Representative traces showing the changes of Ca^2+^ (**A**) or V_m_ (**C**) signals caused by clofilium (50 μM), progesterone (500 nM) or the combination of clofilium and progesterone. Arrows indicate time of stimulation. (**B, D**) Statistical analysis of the amplitude of Ca^2+^ (**B**) or V_m_ (**D**) signals. V_m_ of human sperm was calculated from the fluorescent intensity according to the calibration equation. Control solution contained 0.1% DMSO. Bars indicate the mean ± SD; n = 6 samples. For (**B, D**), repeated measures ANOVA tests were performed (**B**, *p* < 0.0001 and F = 42.02; **D**, *p* = 0.0006 and F = 29.00). Exact *p* values using Bonferroni’s multiple comparisons test were: Ca^2+^ signal (**B**), *p* = 0.0142; V_m_ signals (**D**), *p* = 0.0005. Asterisks indicate statistical significance: *p* < 0.05 (*), *p* < 0.001 (***).

**
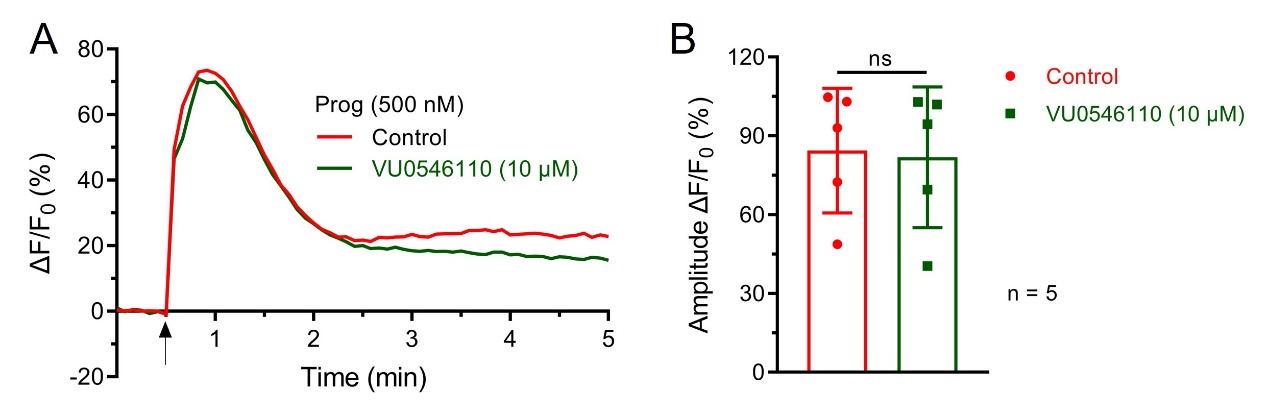
**

**Supplementary Figure 3. VU0546110 had no impact on transient Ca^2+^ response triggered by progesterone.** (**A**) Representative Ca^2+^ signals evoked by progesterone (500 nM). Purified human sperm were pre-treated with 0.1% DMSO (control) or VU0546110 (10 μM) for 30 min. Arrow indicates time of stimulation. (**B**) Statistical analysis of the amplitude of Ca^2+^ signals as shown in (**A**). Bars indicate the mean ± SD, n = 5 samples. Exact *p* value using two-tailed paired *t*-test were 0.2005. ns not significant, *p* ≥ 0.05.


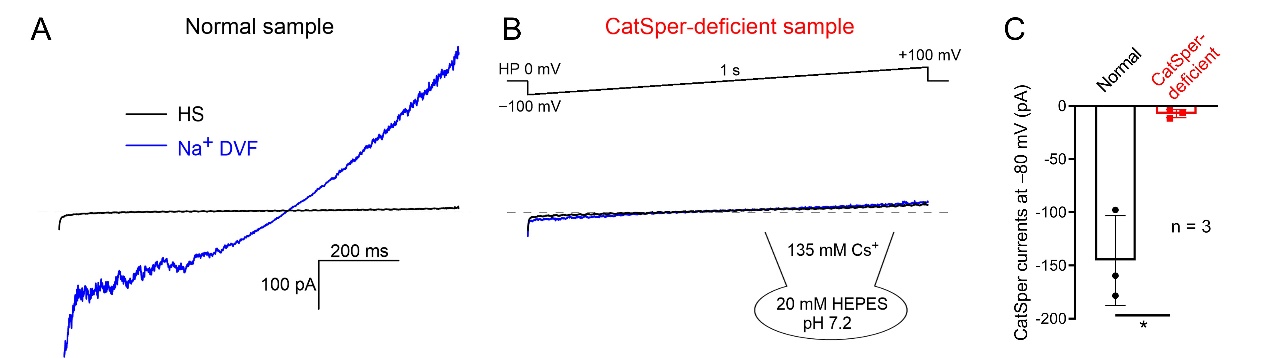


**Supplementary Figure 4. Electrophysiological recordings revealed a specific sperm sample from an infertile patient lack of CatSper function.** (**A, B**) Representative hCatSper current in response to HS or sodium-based divalent free (Na^+^-DVF) solution in a normal (**A**) or CatSper-deficient (**B**) sample. The solution of Na^+^-DVF was used to record the current of monovalent cation mediated by CatSper, which could effectively evaluate the activation of CatSper. A voltage-clamp ramp protocol from −100 mV to +100 mV with a holding potential of 0 mV was used to elicit CatSper currents. (**C**) Statistical analysis of monovalent ion currents of CatSper at −80 mV recorded on human sperm from different normal samples (n = 3 sperm cells) and from the CatSper-deficient sample (n = 3 sperm cells). Bars indicate the mean ± SD, Significant differences using two-tailed unpaired *t*-test with Welch’s correction were observed (*p* = 0.0289). Asterisks indicate statistical significance: *p* < 0.05 (*).


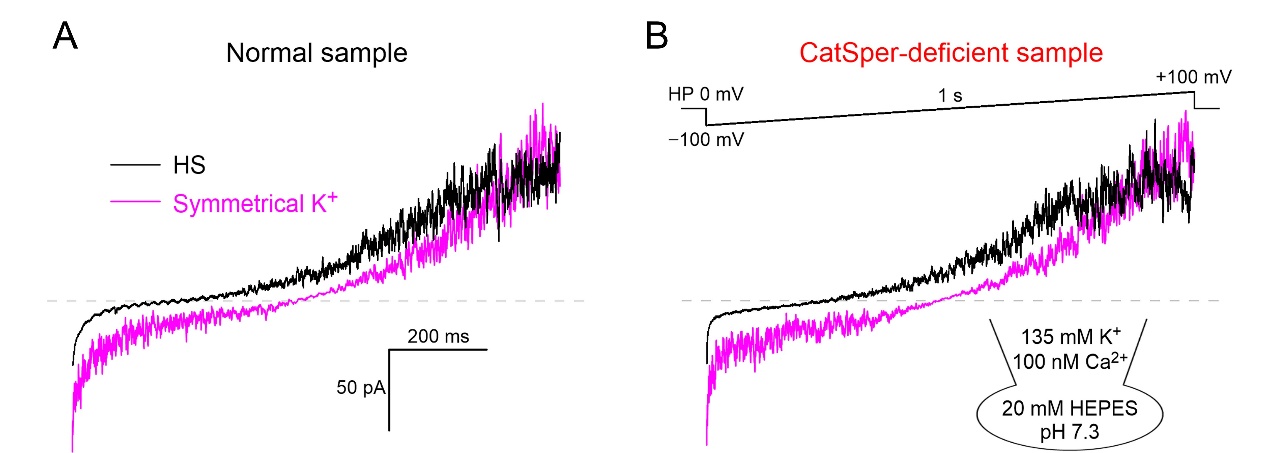


**Supplementary Figure 5. Human KSper currents were basically unaffected in the CatSper-deficient sample.** (**A, B**) Representative hKSper current in response to HS or symmetrical K^+^ (160 mM K^+^) in a normal (**A**) or CatSper-deficient sample (**B**). In order to sufficiently activate hKSper, the pipette solution contained 100 nM Ca^2+^ and the pH was adjusted to 7.3.


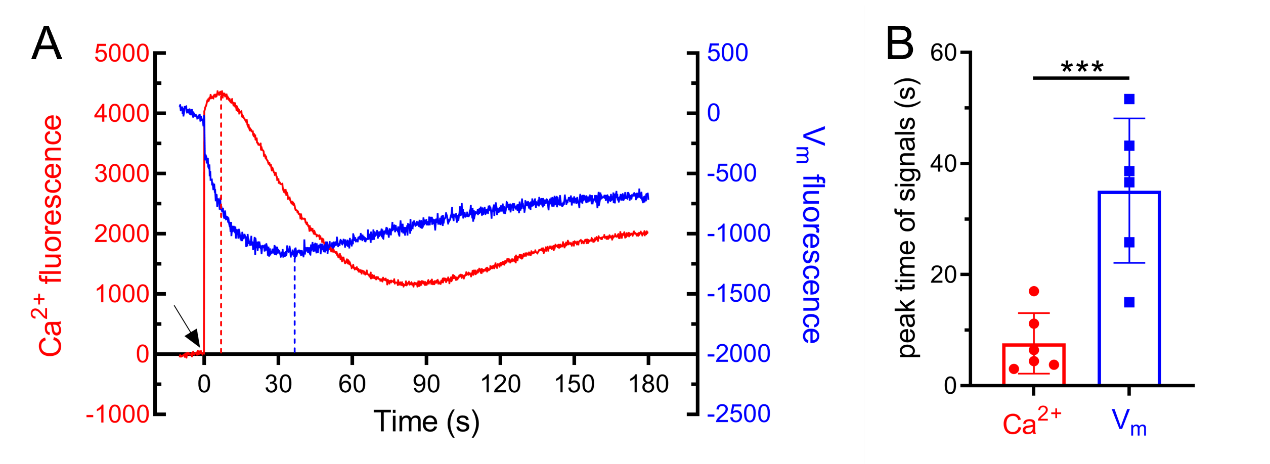


**Supplementary Figure 6. The time course of Ca^2+^ and V_m_ fluorescence in response to progesterone was different**. (**A**) Representative fluorescent traces of Ca^2+^ and V_m_ signals for human sperm loaded with Fluo-4 and DiSC_3_(5). Arrow indicates the stimulation time of progesterone (500 nM). Dash lines indicate the time points for the plateau of signals. (**B**) Mean peak time points of Ca^2+^ and V_m_ signals after the injection of progesterone. Bars indicate the mean ± SD, n = 6 samples. Significant differences using two-tailed unpaired *t*-test were observed (*p* = 0.0007). Asterisks indicate statistical significance: *p* < 0.001 (***).


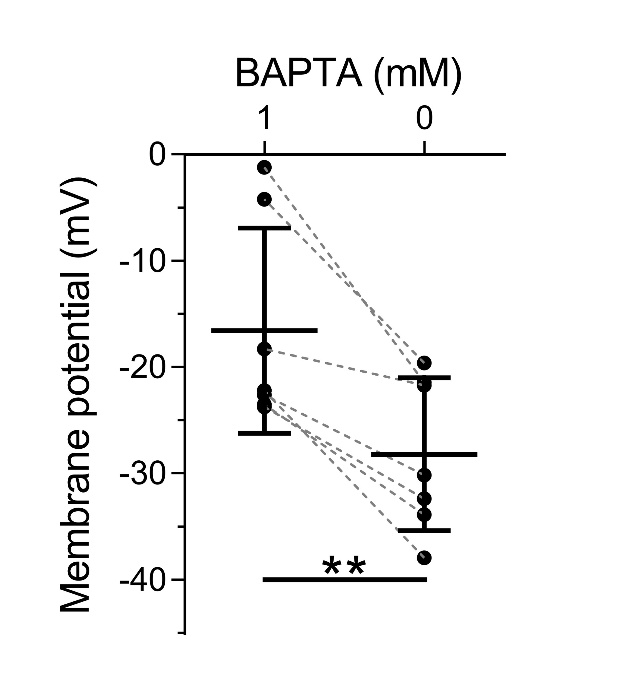


**Supplementary Figure 7. Ca^2+^ ablation in sperm cytoplasm induced the depolarization of V_m_**. Mean V_m_ of human sperm with or without intracellular BAPTA (1 mM) at pH_i_ 6.0. The dashed lines indicate that V_m_ was recorded from the same sample. Bars indicate the mean ± SD. n = 7. Significant differences using two-tailed paired *t*-test were observed (*p* = 0.0018). Asterisks indicate statistical significance: *p* < 0.01 (**).
